# Rapid neck elongation in Sauropterygia (Reptilia: Diapsida) revealed by a new basal pachypleurosaur from the Lower Triassic of China

**DOI:** 10.1186/s12862-023-02150-w

**Published:** 2023-08-31

**Authors:** Qi-Ling Liu, Long Cheng, Thomas L. Stubbs, Benjamin C. Moon, Michael J. Benton, Chun-Bo Yan, Li Tian

**Affiliations:** 1 Hubei Key Laboratory of Paleontology and Geological Environment Evolution, Wuhan Centre of China Geological Survey, Wuhan, 430023 P. R. China; 2 State Key Laboratory of Biogeology and Environmental Geology, China University of Geosciences, Wuhan, Hubei 430078 P. R. China; 3 School of Earth Sciences, Life Sciences Building, Tyndall Avenue, University of Bristol, Bristol, BS8 1TQ UK; 4 School of Life, Health and Chemical Sciences, The Open University, Milton Keynes, UK

**Keywords:** Marine reptile, Eosauropterygia, Mesozoic, Body plan, Nanzhang-Yuan’an Fauna

## Abstract

Neck elongation has appeared independently in several tetrapod groups, including giraffes and sauropod dinosaurs on land, birds and pterosaurs in the air, and sauropterygians (plesiosaurs and relatives) in the oceans. Long necks arose in Early Triassic sauropterygians, but the nature and rate of that elongation has not been documented. Here, we report a new species of pachypleurosaurid sauropterygian, *Chusaurus xiangensis* gen. et sp. nov., based on two new specimens from the Early Triassic Nanzhang-Yuan’an Fauna in the South China Block. The new species shows key features of its Middle Triassic relatives, but has a relatively short neck, measuring 0.48 of the trunk length, compared to > 0.8 from the Middle Triassic onwards. Comparative phylogenetic analysis shows that neck elongation occurred rapidly in all Triassic eosauropterygian lineages, probably driven by feeding pressure in a time of rapid re-establishment of new kinds of marine ecosystems.

**Supplementary Information:**

The online version contains supplementary material available at 10.1186/s12862-023-02150-w.

## Introduction

Marine tetrapods, mainly mammals, occupy various trophic levels in modern marine ecosystems [1]. The situation was very different in the Mesozoic, when a much wider diversity of reptiles occupied similar trophic roles and show evidence of iterative evolution of similar locomotory and feeding modes [1–3]. The similarity of body plan and anatomical characters among different marine tetrapod groups, such as paddle-like limbs or a streamlined body shape, is evidence of morphological convergence in marine adaptation [1, 4, 5]. Various reptile clades invaded the ocean during the Mesozoic, among which Sauropterygia, Ichthyopterygia, and marine clades of Testudinata, are the longest-lasting clades. Both ichthyosaurs and sauropterygians have their first known occurrences in the Early Triassic, while the earliest fossil record of a marine turtle comes from the Late Triassic [6–9]. Ichthyosaurs evolved large body size rapidly and adopted a thunniform body plan convergent with cetaceans [10, 11]. Sea turtles exist in the modern ocean, but sauropterygians have no living analogue despite the evident success of their body plan body plan in the Mesozoic, especially the extremely long-necked plesiosaurian sauropterygians. A similar body plan could be seen in some other Mesozoic marine reptiles, like tanystropheids, but no living tetrapod has a neck that is longer than its trunk, except giraffes on land and some birds [1, 12].

Sauropterygia diversified and became widespread during the Early-Middle Triassic, but only the long-necked pelagic lineage survived past the Late Triassic. Compared to abundant sauropterygians in the Middle Triassic, reports from the Lower Triassic are limited to a few incomplete specimens, but several clades became long-necked postdating the Late Triassic. While *Tanystropheus* and *Dinocephalosaurus* increased the length of each vertebra to attain a long neck, neck length in Sauropterygia shows a different pattern, mostly achieved by increasing the number of vertebrae [13]. Elasmosauroids from the Cretaceous are well known for their extremely elongated necks, sometimes with over 70 cervical vertebrae [14, 15]. The neck elongation in Triassic eosauropterygians is less extreme, but the origin and rate of acquisition of this feature is obscured by the absence of good fossils of basal taxa. Although a handful of eosauropterygian fossils have been reported in the Early Triassic, most of them are too incomplete to allow precise measurements of head and neck, such as the known specimens of *Majiashanosaurus discocoracoidis* and *Lariosaurus sanxiaensis* [9, 16, 17]. Nevertheless, the recently reported [18] nearly complete specimen of *Hanosaurus hupehensis*, as well as some *Corosaurus alcovensis* specimens [19–21], provide the desired measurements, but good quality basal pachypleurosaurids are required to further explore the earliest stages of the neck-elongation trend among different eosauropterygian clades.

Here we report and describe two specimens of a new eosauropterygian taxon from the Early Triassic Nanzhang-Yuan’an Fauna (NYF), Hubei Province, P. R. China, which aid in mapping the evolution of neck elongation in Eosauropterygia. The newly discovered WGSC V 1901 is nearly complete, providing vital material to quantify the body plan of early marine reptiles, such as neck length/trunk length ratio, and to explore the history of neck elongation in Eosauropterygia.

## Results

### Geological setting

**Fig. 1:**
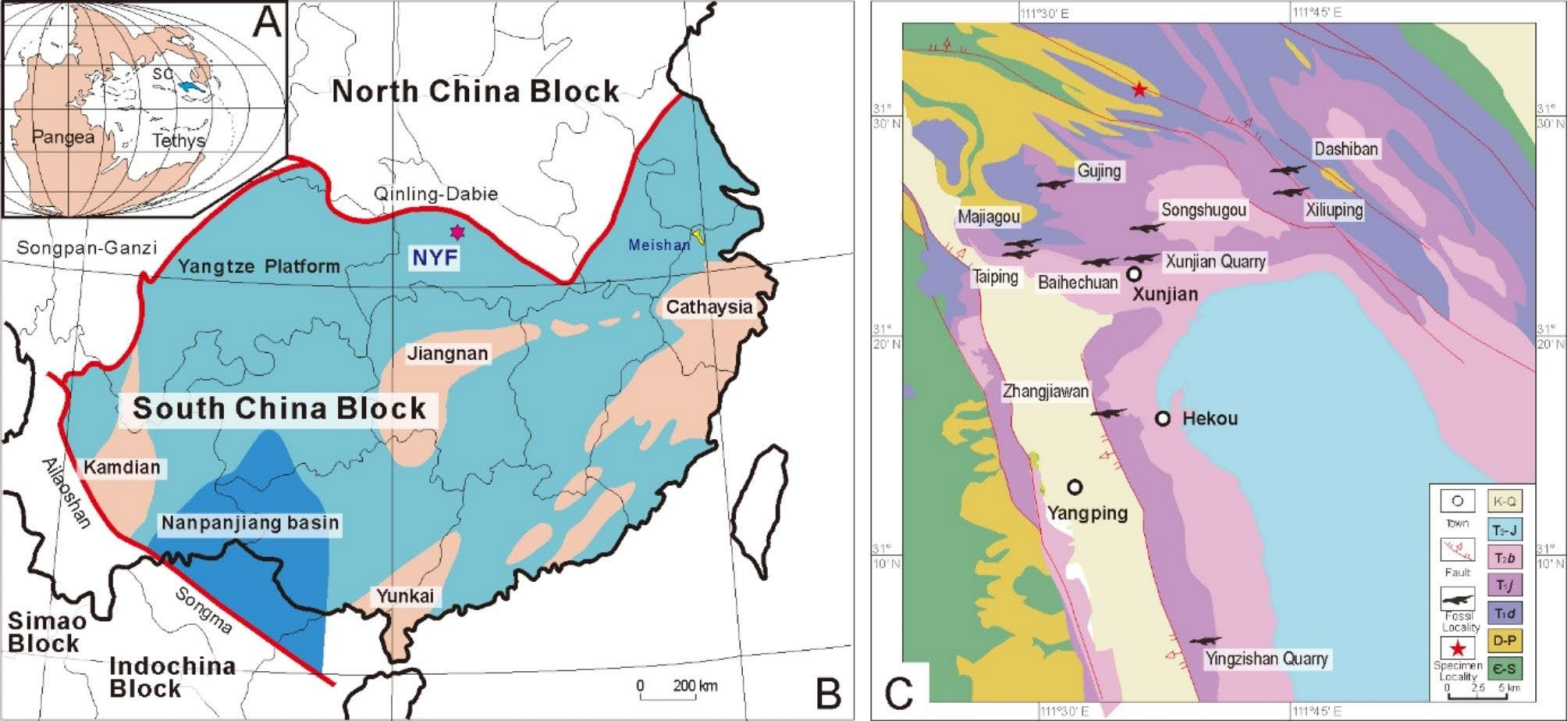
Summary geological maps. **(A)** The general location of the South China block in the Early Triassic (modified from Benton et al. [24]). **(B)** Tectonic map showing major blocks of South China [24], with the site of Nanzhang-Yuan’an Fauna (NYF). **(C)** Simplified geological map of Nanzhang and Yuan’an counties with distributions of Triassic marine reptiles (modified from Li et al. [16] and Yan et al. [22]). ‘Old land’ is coloured orange, shallow seas light blue, and deep marine basins dark blue in **A**, **B**. Abbreviations: Є-S, Cambrian-Silurian; **D-P**, Devonian-Permian; **T**_**1d**_, Daye Formation, Lower Triassic; **T**_**1j**_, Jialingjiang Formation, Lower Triassic; **T**_**2b**_, Badong Formation, Middle Triassic; **T**_**3**_**-J**, Upper Triassic-Jurassic; **K-Q**, Cretaceous-Quaternary.

The NYF occurs at several carbonate outcrops between Nanzhang and Yuan’an counties, Hubei Province, P. R. China (Fig. 1). Fossiliferous beds with abundant marine reptile skeletons and bone fragments mainly occur in the upper part of Member II of the Jialingjiang Formation, comprising laminated micritic carbonates [22]. A zircon U-Pb age of 247.8 ± 1.2 Ma [22, 23] from the volcanic tuff at the top of Member II indicates its age is Spathian (late Olenekian; Early Triassic). The sediments indicate a restricted shallow water or lagoonal environment, coinciding with the palaeogeography of the Yangtze Block in the Triassic [22–24].

### Systematic palaeontology

Superorder Sauropterygia Owen, 1860 [25].

Order Eosauropterygia Rieppel, 1994 [26].

Family Pachypleurosauridae Nopcsa, 1928 [27].

Genus *Chusaurus*, gen. nov.

(Figs. 2 and 3)

**Fig. 2:**
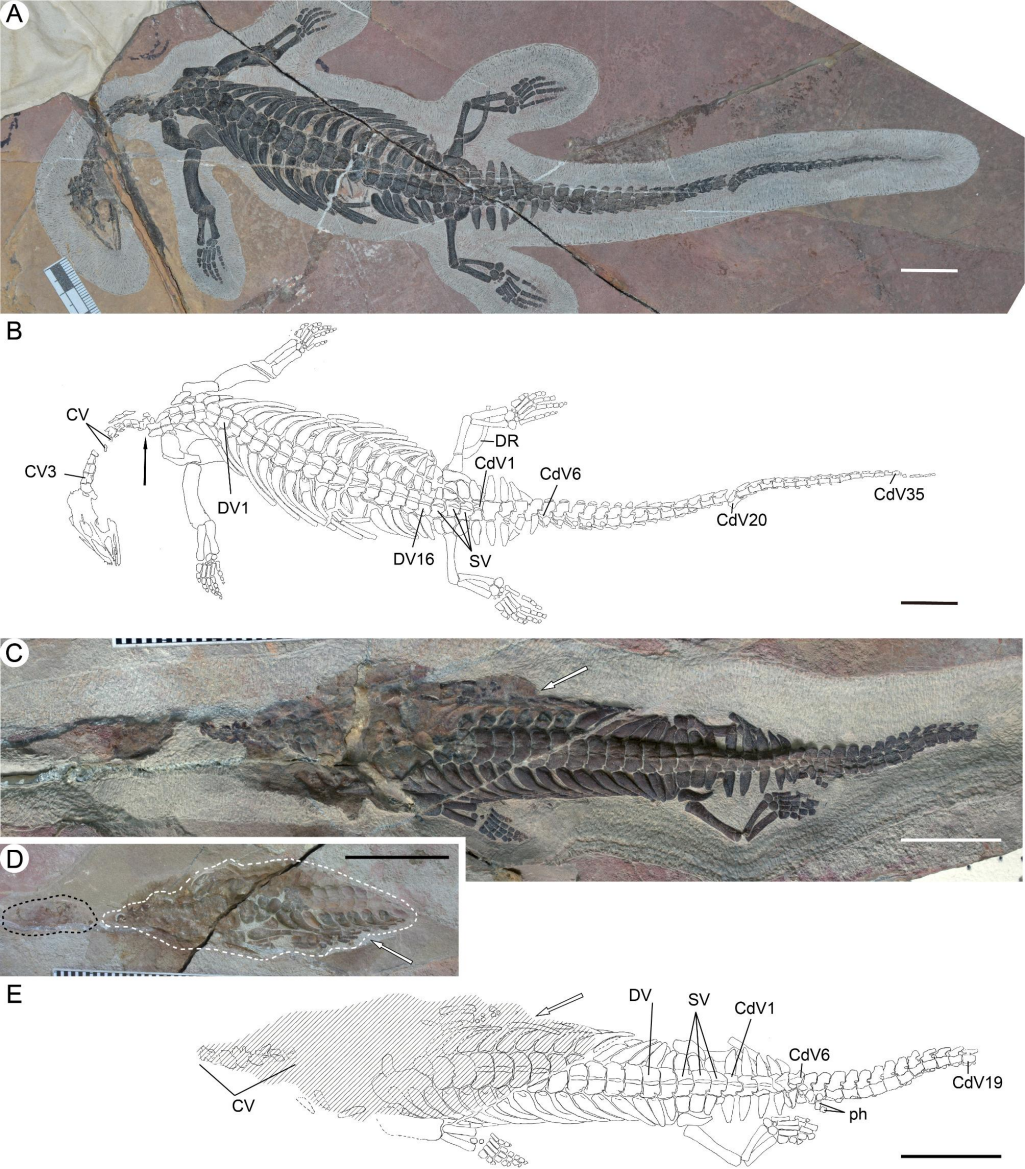
The two specimens of *Chusaurus xiangensis* gen. et sp. nov. **(A)** Photograph of WGSC V 1901, mostly in dorsal view. **(B)** Interpretive drawing of WGSC V 1901. **(C)** Photograph of WGSC V 1702, dorsally exposed. **(D)** Mould and part of the skeleton from the counterpart of WGSC V 1702. **(E)** Interpretive drawing of WGSC V 1702. The black arrow in **B** indicates the abrupt inversion of the cervical vertebral column. Skull elements are marked by a black dashed line in **D**, and shadow in **E**, and represent the shape of the mould in **D** marked by a white dashed line. White arrows indicate the corresponding parts in **C**-**E**. **Abbreviations: CdV**, caudal vertebra; **CV**, cervical vertebra; **DR**, dorsal rib; **DV**, dorsal vertebra; **ph**, phalanx; **SV**, sacral vertebra. Scale bar = 2 cm

**Fig. 3:**
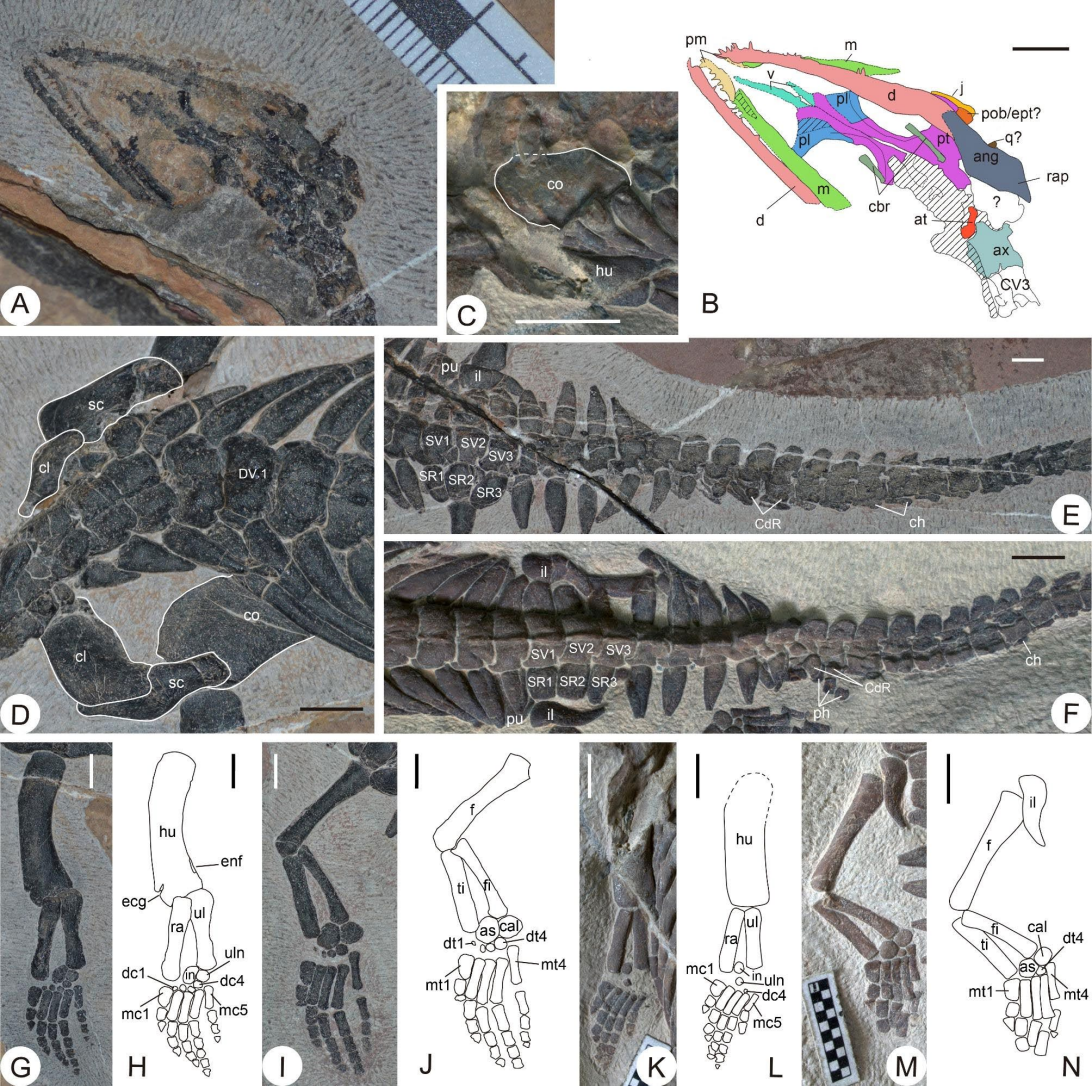
Selected details of the two specimens. **(A)** Skull of the holotype, ventrally exposed. **(B)** Interpretative drawing of **A**. **(C)** Coracoid of WGSC V 1702. **(D)** Pectoral region of the holotype in dorsal view. **(E)** Pelvic and anterior caudal region of the holotype in dorsal view. **(F)** Pelvic and anterior caudal region of WGSC V 1702 in dorsal view. **G-J.** Forelimb and hindlimb of the holotype in dorsal view. **K-N.** Forelimb and hindlimb of WGSC V 1702 in dorsal view. Dashed line represents conjectural borderline. Shade in **B** represents unidentified elements. **Abbreviations: ang**, angular; **as**, astragalus; **at**, atlas; **ax**, axis; **cal**, calcaneum; **cbr**, ceratobranchial; **CdR**, caudal rib; **ch**, chevron; **cl**, clavicle; **co**, coracoid; **CR**, cervical rib; **d**, dentary; **dc**, distal carpal;**dt**, distal tarsal; **DV**, dorsal vertebra; **ecg**, ectepicondylar groove; **enf**, entepicondylar foramen; **f**, femur; **fi**, fibula; **hu**, humerus; **il**, ilium; **in**, intermedium; **j**, jugal; **m**, maxilla; **mc**, metacarpal; **mt**, metatarsal; **ph**, phalanx; **pl**, palatine; **pm**, premaxilla; **pob/ept?**, postorbital or ectopterygoid; **pt**, pterygoid; **pu**, pubis; **ra**, radius; **rap**, retroarticular process; **sc**, scapula; **SR**, sacral rib; **SV**, sacral vertebra; **ti**, tibia; **ul**, ulna; **uln**, ulnare. **v**, vomer. Scale bar in **B-L**, **N** = 5 mm. Scale division in **A, M** = 1 mm

### Type species

*Chusaurus xiangensis*, gen. et sp. nov.

### Distribution

Olenekian (Early Triassic), South China.

### Diagnosis

As for type species, the only known species in this genus.

*Chusaurus xiangensis*, gen. et sp. nov.

### Holotype

WGSC (Wuhan Centre of China Geological Survey) V 1901, a nearly complete skeleton lacking some cranial and cervical vertebral elements (Fig. 2A–B).

### Referred specimen

WGSC V 1702, an articulated skeleton preserving part of the cranial and incomplete postcranial regions, lacking some cervical, shoulder girdle, appendicular, and posterior caudal elements (Fig. 2C–E).

### Type locality and horizon

Songshugou Village, Nanzhang County, Xiangyang City, Hubei Province, P. R. China. Member II of the Jialingjiang Formation; Spathian, late Olenekian, Early Triassic.

### Etymology

The generic name is derived from the Chu Kingdom in Chinese history, which dominated around the area of the fossil site. The specific epithet is derived from “Xiang”, referring to Xiangyang City where the fossil was discovered.

### Diagnosis

A small-sized pachypleurosaurid eosauropterygian with at least 17 cervical, 16 dorsal, 3 sacral, and about 40 caudal vertebrae; neck length about half the trunk length; pterygoid flanges well developed and longitudinally oriented; anteriormost caudal vertebral neural spines obviously heightened compared to presacral and sacral vertebrae; dorsal and anterior caudal ribs highly pachyostotic with expanding proximal end; posteriormost caudal ribs shortened and round; chevron well developed in the caudal region; clavicle short and stout; scapula elongate and without broadly expanded anterior portion; scapular blade is short and robust; coracoid without concave anteromedial margin; iliac blade well developed with a posterior process; little interspace between the manual and pedal digits; the proximal phalanges short and flattened; 6 maximum carpal and tarsal ossifications; manual phalangeal format 2-3-4-5-3; pedal phalangeal format 2-3-4-5-4.

### Brief description

Salient morphological features are summarized here, with additional detailed description provided in the Supplemental Information. The skull is ventrally exposed in the holotype, but the preservation is rather poor (Fig. 3A–B). There are indications of anterior rostral narrowing in the premaxillary portion. Both the palatine and the pterygoid develop a lateral flange. The pterygoid is shattered along the flange, exposing the orbit in ventral view. The anteriormost dentary teeth are comparatively large, with a count of 5–6. The orbit is quite large, as in many other small pachypleurosaurs.

In the holotype, the cervical vertebral column shows an unnatural overturn between the fifth and likely the ninth vertebra (Fig. 2B). The vertebrae anterior to this overturn are ventrolaterally exposed, while more posterior elements are dorsally exposed. There are at least 17 cervical vertebrae in the holotype, 16 dorsal vertebrae, 3 sacral vertebrae, and about 40 caudal vertebrae (CdV). WGSC V 1702 shows the same count of sacral vertebrae as the holotype, whereas the presacral and caudal vertebral elements are incomplete (Fig. 3E–F). As seen in both specimens, the neural spine of the dorsal region is very low, however, the height increases in the sacral region and anteriormost caudal region distinctly, reaching its maximum height in the caudal vertebrae CdV2–10 and decreasing posteriorly (Fig. 3E–F). A similar trend in neural spine height is observed in many pachypleurosaurids [28]. The accessory articulation between the dorsal neural arches (Fig. 3D, F) that is widely found in pachypleurosaurids [29] is also present in *C. xiangensis*, though it is not obvious. Thickening of the curving dorsal ribs, which is indicative of pachyostosis, is obvious in both specimens, as in many other pachypleurosaurs. A groove could be recognized in WGSG V 1702 at the proximal end of the dorsal ribs, which represents the articulation facet with the corresponding vertebra (Fig. 2C, E). Chevrons are developed in the caudal region (Fig. 3E–F). The first chevron appears at CdV5 in the holotype, while in the WGSG V 1702 the chevron could be recognized from CdV8 owing to its preservation. Chevron size decreases along the caudal vertebral column, disappearing in CdV28 in the holotype.

Based on WGSC V 1702, the form of the coracoid in *C. xiangensis* is like that in *Majiashanosaurus discocoracoidis* (Fig. 3C–D) [9], and lacks the stronger waisted outline seen in Middle Triassic pachypleurosaurs like *Keichousaurus hui*, *Dianmeisaurus gracilis*, and *Diandongosaurus acutidentatus* [30–32]. The humerus is curved, with a convex anterior margin and a concave posterior margin (Fig. 3G–H, K–L), a pattern characteristic of Sauropterygia [33]. Six carpal ossifications are observed in both manus from the holotype (Fig. 3G–H), namely two large elements, the intermedium and the ulnare, and four small distal carpals, while WGSC V 1702 only has the three carpal ossifications (Fig. 3G–H, K–L). This suggests that *C. xiangensis* bears at least three carpal ossifications. The difference in counting might be caused by taphonomic processes that disarticulated the distal carpals of WGSC V 1702. The manual digits are deflected towards the ulnar side, and the interdigital space between digits 4 and 5 is the widest in the holotype, whereas in WGSC V 1702 all digits are tightly articulated. In general, the phalanges are stout and massive compared to other eosauropterygians.

Viewed dorsally, the ilium develops a pointed posterior process, different to known pachypleurosaurid taxa, which mostly have poorly developed dorsal blades [32, 34]. Six tarsal ossifications are preserved in both hindlimbs of the holotype: the astragalus, the calcaneum, and four distal tarsals, while the referred specimen preserves only three distal tarsals (Fig. 3I–J, M–N). Fewer carpal and tarsal elements probably suggest an earlier ontogenetic stage of WGSC V 1702 than the holotype, though taphonomic effects cannot be excluded. The six tarsal ossifications in the holotype are a relatively high number among pachypleurosaurs, as the count is usually two or three [17, 21, 32, 35, 36]. Notably, even under different preservation conditions, the interspaces between manual and pedal digits are rather limited in both specimens, suggesting a natural morphological character.

### Evidence for a new taxon

The anatomical characters of the limbs and dorsal vertebrae suggest that WGSC V 1901 and WGSC V 1702 are a new taxon rather than examples of *Keichousaurus yuananensis*, which was the first pachypleurosaur discovered from NYF in 1965 [37]. Unfortunately, the original report of *K. yuananensis* is rather poor; the holotype is just a moulage without clear illustrations, and the whereabouts of the holotype are unknown. According to the original description by Young [37], *K. yuananensis* resembles *K. hui* in the plate-like ulna which is shorter than the radius, but the length and width of the two bones are equal in *Chusaurus*. Further, the shape of the coracoid in *K. yuananensis* resembles *K. hui* [37], with a waisted outline, while the counterpart in *C. xiangensis* shows a smooth anteromedial margin (Fig. 3C–D). In *C. xiangensis*, all the dorsal ribs have expanded proximal ends, but only the anterior dorsal ribs of *K. yuananensis* show this character [37]. Moreover, *K. yuananensis* has slender rod-like phalanges [37], differing distinctly from *C. xiangensis*, whose phalanges is short and broadened (Fig. 3G–N).

*C. xiangensis* also show differences from other known pachypleurosaurid taxa. It shows a broadened clavicle that lacks the anterolateral process in *Diandongosaurus* [38] and *Dianmeisaurus* [32], or anterolateral expansion in *Dawazisaurus* [8] and *Dianopachysaurus* [34]. In addition, many pachypleurosaurs have hourglass-shaped or rod-like slender metacarpals and metatarsals, including *K. hui* [29], *Dactylosaurus* [39], and *Majishanosaurus* [9], whereas *C. xiangensis* has stout and massive ones. *C. xiangensis* also has highly pachyostotic dorsal ribs without constriction near the proximal ends, differing from many other pachypleurosaurid taxa (e.g. *Anarosaurus* [40], *Serpianosaurs* [28], *Honghesaurus* [41], and *Prosantosaurus* [42]). Though poorly preserved, the snout of *C. xiangensis* is neither the rounded shape in *Panzhousaurus* [43], nor tapering like *Luopingosaurus* [44]. Thus, we consider that WGSC V 1901 and WGSC V 1702 are a new taxon from their combination of these morphological characters.

### ***Chusaurus xiangensis*** gen. et sp. nov. is a basal pachypleurosaur

**Fig. 4:**
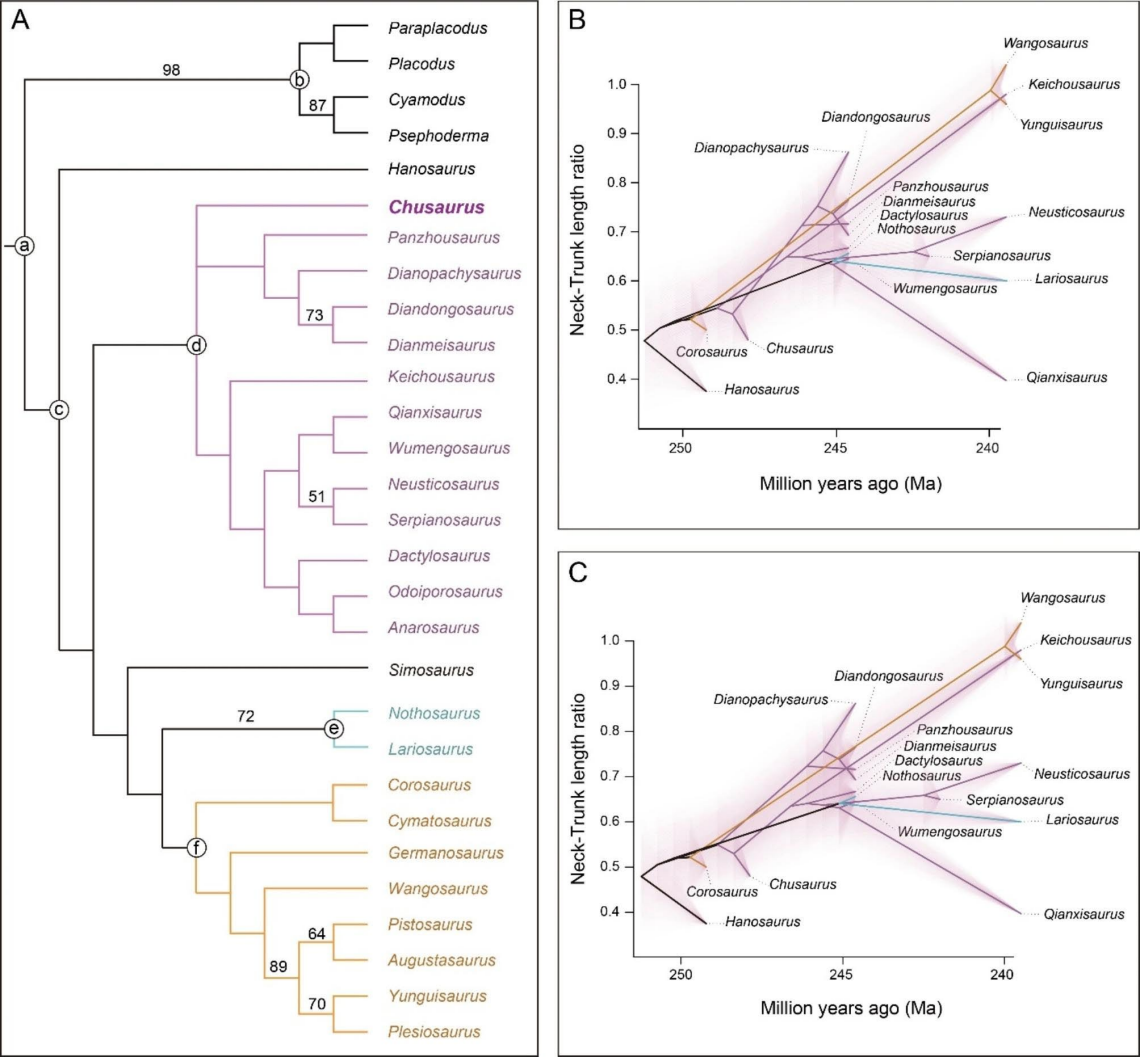
Phylogenetic position of the new taxon and phenograms of relative neck length in selected Triassic eosauropterygians. **(A)** Strict consensus of four most parsimonious trees (TL = 534, CI = 0.352, RI = 0.613). Bootstrap values ≥ 50% are labelled. Taxa are marked by different colours. **(B)** Phenograms of neck-trunk length ratio (*Chusaurus* as sister group to the clade including *Panzhousaurus*). **(C)** Phenograms of neck-trunk length ratio (*Chusaurus* as sister group to the clade including *Keichousaurus*). **Clades: a.** Sauropterygia; **b**. Placodontia; **(c)** Eosauropterygia; **(d)** Pachypleurosauridae; **(e)** Nothosauridae; **(f)** Pistosauroidea

We identify *Chusaurus xiangensis* gen. et sp. nov. as a relatively basal member of Pachypleurosauridae based on our cladistic analysis (Fig. 4A; see Fig. S3 for the full version of the tree). Though, regrettably, the relationships within Pachypleurosauridae have not been completely resolved by our phylogenetic analysis, *C. xiangensis*, gen. et. sp. nov. is regarded as a basal pachypleurosaur, though the polytomy suggests its relationship with two clades, one including *Keichousaurus* and the other including *Panzhousaurus*, is currently unknown.

Morphological trait analysis confirms this phylogenetic placement. The coracoid has a smooth anteromedial margin without a strongly waisted outline, a transition from the rounded coracoid of *Hanosaurus hupehensis* and *Majiashanosaurus discocoracoidis*, both of which are Early Triassic sauropterygians [9, 17] to the derived pachypleurosaurs with “coracoid strongly waisted” [30]. Meanwhile, the iliac blade of *C. xiangensis*, gen. et sp. nov. is well developed, whilst pachypleurosaurs from the Middle Triassic usually have a reduced or even absent iliac blade, like *Serpianosaurus*, *Neusticosaurus*, *Wumengosaurus*, *Dianopachysaurus* and *Panzhousaurus* [28, 34, 35, 43, 45] (see Supplemental Information, Fig. S2). More importantly, *C. xiangensis* already develops a longer humerus than femur, which indicates further aquatic adaptation compared to *Hanosaurus* [3], the most basal taxon with comparatively short forelimbs [18]. Yet the high level of pachyostosis in *C. xiangensis* identifies it as a shallow water, even semiaquatic animal [46].

## Discussion

### Neck length macroevolution in Eosauropterygia

The Early Triassic was a time of rapid evolution of life in the oceans, following the devastating end-Permian mass extinction, marked especially by the appearance of new animals and new modes of life [2, 4, 24]. In particular, new benthic groups such as bivalves provided food for new predatory gastropods, malacostracans, echinoids and fishes. These in turn provided food for durophagous fishes and marine reptiles and macropredatory reptiles that fed on fishes and other reptiles [47]. Long necks likely evolved as an adaptation to snapping rapidly at the faster swimming fishes of the new ecosystems or dipping for benthic prey in murky seabed sediments [48].

Phenograms of the neck-trunk length ratio (Fig. 4B–C) show a rapid expansion in the first five million years of eosauropterygian evolution, as the ratio increases from 0.4 to 0.9. This is followed by later but slower expansion in the Middle to early Late Triassic when ratios between 0.9 and > 1 occurred for the first time. Our analyses show that the basal eosauropterygian *Hanosaurus* (ratio = 0.376), the basal pistosauroid *Corosaurus* (ratio = 0.500), and *C. xiangensis* (ratio = 0.480) all have relatively short necks compared to the derived taxa in their clades, and lengthening is seen independently in both pistosauroids and pachypleurosaurs.

The long-necked plesiosaurian clades, first occurring in the latest Triassic [49], evolved from Early–Middle Triassic short-necked eosauropterygian predecessors. The number of cervical and dorsal vertebrae is the key factor that influences the relative length of neck in derived plesiosaurians [13, 15], a similar pattern shared by pachypleurosaurids and basal pistosauroids. *Hanosaurus* (ratio = 0.376), the most basal taxon, has a relative long trunk as an ancestral body plan, with 25 dorsal and 15 cervical vertebrae [18]. The ratio increases to 0.480 in *Chusaurus*, from the change of vertebral number (17 cervical and 16 dorsal). When it comes to *Keichousaurus* (ratio = 0.980), the number of cervical vertebrae increases to 25 with 19 dorsal [29]. This is in line with the pistosauroids, as the ratio reached 0.96 by the end of the Middle Triassic, equivalent to the long neck of *Keichousaurus*. Though the incomplete specimens of the earliest nothosaurid *Lariosaurus sanxiaensis* cannot provide enough information to track the neck length, the Middle Triassic taxa share a similar neck length in the clade (Supplemental Information, Table S1).

Thus, a relatively short neck is most probably an ancestral character in Eosauropterygia, and neck elongation originated independently in Triassic pachypleurosaurid and pistosauroid clades, but the short neck reappeared in the derived pliosaurians during the Jurassic and polycotylids in the Cretaceous, in which a long head and large body evolved iteratively as adaptations to different niches [50]. The ratio varies greatly among derived plesiosaurian clades (Table S1), often deciphered as homeotic changes [13, 51]. In addition, evolutionary variability in the vertebral column appears in pachypleurosaurids as well. For example, *Qianxisaurus chajiangensis* [52] from the Middle Triassic Xingyi fauna developed a short neck with 18 cervical and 28 dorsal vertebrae, whose length/trunk length ratio is 0.397, even lower than in *C. xiangensis*, gen. et sp. nov (Fig. 4B–C).

### Convergence of neck elongation among tetrapods

Extreme long necks were not unique to eosauropterygians, but occurred also in some marine archosauromorph taxa, such as *Tanystropheus* and *Dinocephalosaurus* [53, 54]. However, their neck length depends on elongation of each cervical vertebra rather than increasing the number. These two genera show various characters as adaptations for ocean life, such as viviparity in *Dinocephalosaurus* [55], but they did not give rise to a larger clade and were extinct before the end of the Triassic [54].

Neck elongation evolved independently numerous times among extinct marine reptiles, dinosaurs and pterosaurs, and modern mammals and birds, and is generally explained by selection pressure related to feeding strategy [12, 56, 57]. The classic explanation for neck elongation in the giraffe is as an adaptation for browsing high in the tree canopy [12]. Similarly, the long necks of the giant terrestrial sauropod dinosaurs are explained as adaptations to feed high, and at different heights, in the tree canopy [12, 56, 57].

We suggest here that variations in the neck length of eosauropterygians may reflect different feeding strategies as well: a typical short-necked pliosauromorph is regarded as the apex predator in Mesozoic marine ecosystems, while some of the long-necked plesiosaurs were more likely to be mesophagous, with their long neck enabling them to pursue smaller prey such as fast-moving fishes or benthic animals, like bivalves on the seabed [48, 50, 58]. In these cases, the long, flexible neck with many cervical vertebrae, would enable the hunter to flip its head faster in pursuit of fishes than by moving the whole body, or to search for food over a wide area on the murky seabed without constantly moving the body. As for pachypleurosaurids, they are believed to have fed on invertebrates and small fishes in shallow waters, adopting the suction strategy like aquatic chelonians [47]. Therefore, the neck elongation might have contributed to this ‘fast-strike’ feeding strategy, helping to capture the prey in an ambush.

## Methods

### Phylogenetic analysis

To resolve the phylogenetic position of the new specimens, a cladistic analysis was conducted using the morphological data matrix in Lin et al. [59] with the new taxon added. We excluded taxa with coding < 0.33 of characters (*Majiashanosaurus* and *Bobosaurus*). The matrix comprises 34 taxa and 148 morphological characters (see Supplemental Information). The matrix was generated using Mesquite version 3.61 [60]. The phylogenetic analysis was performed using TNT version 1.5 [61], employing a traditional search, with 1000 random addition sequences, 10 trees saved per replication and TBR swapping. Then bootstrap values were computed in PAUP*4.0a169 [62] with 100 replicates for resampling. The phylogenetic analysis recovered four most parsimonious trees, each with length 534 steps. *Chusaurus xiangensis* is identified as one of the most basal taxa within a monophyletic Pachypleurosauridae; Nothosauridae and Pistosauroidea are situated within a monophyletic Eusauropterygia as well, but *Germanosaurus* and *Simosaurus* are not within Nothosauridae, which is different from the finding of Lin et al. [59].

### Ancestral state estimation

We estimated ancestral states for neck length/trunk length ratios in R, using the package phytools and the fastAnc and phenogram functions [63]. Our consensus topology was cropped to include just Triassic eosauropterygians, and the tree was then geologically time-calibrated using occurrence dates and the function timePaleoPhy in the R package paleotree [64]. Prior to ancestral state estimation we randomly resolved the consensus tree, which resulted in two main topologies, with *Chusaurus xiangensis* belonging to either of the two pachypleurosaurid sub-clades. The time-calibrated trees were plotted as phenograms reflecting the known neck:trunk length ratios for each taxon and with estimated ancestral states incorporating uncertainty through simulations. The first and last appearance data (FAD and LAD) are based on Gutarra et al. [56] (see Supplemental Information).

### Electronic supplementary material

Below is the link to the electronic supplementary material.

Supplementary Material 1

## Data Availability

All data generated or analysed during this study are included in this published article and its online supplementary information file. The specimens of *Chusaurus xiangensis* are stored in the museum of the Wuhan Center of China Geological Survey (Wuhan, Hubei Province, P. R. China) under the collection numbers WGSC V 1901 and WGSC V 1702.
