# Supplementary material for: Rapid neck elongation in Sauropterygia (Reptilia: Diapsida) revealed by a new basal pachypleurosaur from the Lower Triassic of China
Source: BMC Ecol Evol. 2023 Aug 31;23:44. doi: 10.1186/s12862-023-02150-w (PMC10469986; doi:10.1186/s12862-023-02150-w)
Supplement: Supplementary file 1 — Supplementary Material 1 [file 12862_2023_2150_MOESM1_ESM.pdf]

Contents

Supplemental Section S1. Detailed Description 1

Supplemental Fig. S1. Skull and selected postcranial parts of holotype 6

Supplemental Fig. S2. Skull and selected postcranial parts of holotype 7

Supplemental Fig. S3. Strict consensus of 4 most parsimonious trees 8

Supplemental Table S1. Neck length/trunk length ratio of selected eosauropterygians 9

Supplemental Table S2. Selected measurements (in mm) of WGSC V 1901 and WGSC V 1702 ... 12

Supplemental Table S3. Neck length/trunk length ratio for R 14

Supplemental Dataset S1. Matrix for phylogenetic analysis 15

References 20

Section S1. Detailed Description

Skull and dentition. The skull is ventrally exposed in the holotype, and yet the preservation is rather poor (Fig. 3A–B). There are indications of anterior rostral narrowing in the premaxillary portion. The premaxilla is partly exposed, bearing about eight small teeth. The maxilla is long, with teeth on its anterior part, while the posterior part is missing. The jugal is partly exposed and articulated to the pterygoid. The vomer is poorly preserved, articulated to the pterygoid posteriorly, and defines the medial margin of the internal naris. The palatine is articulated to the pterygoid medially, forming the posterior margin of the internal naris and the anterior margin of the orbit. The pterygoid is paired, meeting along the midline. Furthermore, the pterygoid develops an anterolateral process, defining the posterolateral margin of the internal naris and separating the palatine and vomer. Both the palatine and the pterygoid develop a lateral flange. The pterygoid is shattered along the flange, exposing the orbit in ventral view. The dentary forms most of the mandible, bearing small teeth like the maxillary ones, restricted to the portion anterior to the orbit. The anteriormost dentary teeth are comparatively large, with a count of 5–6. The angular is articulated to the dentary posteriorly, and the retroarticular process is well developed. The ceratobranchial is a small rod-like element ventral to the pterygoid. The suborbital fenestra is quite large, as in many other small pachypleurosaurs. Other palatal elements posterior to the pterygoid could not be identified due to the taphonomic condition.

Axial skeleton. In the holotype, the cervical vertebral column shows an unnatural overturn between the fifth and likely the ninth vertebra (Fig. 2B). The vertebrae anterior to this overturn are ventrolaterally exposed, while more posterior elements are dorsally exposed. In the holotype, there are at least 17 cervical vertebrae, 16 dorsal vertebrae, 3 sacral vertebrae, and about 40 caudal vertebrae (CdV). WGSC V 1702 shows the same count of sacral vertebrae as the holotype, whereas the presacral and caudal vertebral elements are incomplete (Fig. 2C). A ridge develops on the ventral surface of the cervical vertebral centrum (Fig. S1). As seen in both specimens, the neural spine of the dorsal region is very low, but the height increases in the sacral region and anteriormost caudal region distinctly, reaching its maximum height in the caudal vertebrae CdV2–10 and decreases posteriorly (Fig. 3E–F). A similar trend in neural spine height is observed in many pachypleurosaurids, such as *Serpianosaurus mirigiolensis*: the dorsoventrally tallest of the whole vertebral column are the first

three to five caudals, related to aquatic propulsion[1]. However, the neural spine height increase in *S. mirigiolensis* initiates in the posteriormost dorsal region[1], whereas the trend starts in the sacral region in *C. xiangensis* (Figs 3E–F). The dorsal neural arch does not have the butterfly-shaped form in dorsal view typical of pachypleurosaurids in the dorsal region[2, 3], due to the widened diapophysis as well as shorter pre- and postzygapophyses. Nevertheless, the accessory articulation of the dorsal neural spine (Fig. S1) in pachypleurosaurids exists in *C. xiangensis*, though it is not obvious[4]. In the laterally-exposed caudal vertebrae, the articulation facets of pre- and postzygapophyses are horizontal (Fig. S1), like some other pachypleurosaurs, e.g. *Odoiporosaurus teruzzii*, *Keichousaurus hui* and *Serpianosaurus mirigiolensis*[1, 2, 4].

The cervical ribs are identified by their pointed distal ends, while the dorsal ribs have cylindrical, blunted distal ends (Fig. S1), as typical dorsal ribs usually have flat distal ends in other pachypleurosaurs, like *Anarosaurus*[5]. Consequently, CR17 is identified as a cervical rib, even though it is elongated. The size of the cervical ribs increases along the vertebral column. The anterior cervical ribs develop an anterior process, which disappears in the posterior cervical ribs from the antepenultimate. Pachyostosis of the curving dorsal ribs is obvious in both specimens, as in many other pachypleurosaurs, but with more expanded proximal ends resembling *Lariosaurus sanxiaensis*[6]. A groove could be recognized in WGSC V 1702 at the proximal end of the dorsal rib, as an articulation to the vertebral column (Fig. 2C, E). The articulation facets of the dorsal ribs on the vertebral centrum face posteriorly. The lengths of the dorsal ribs (DR) decrease rapidly from DR13 to the level of the sacral ribs. The three sacral ribs are identified according to a flattened distal end meeting the ilia and do not co-ossify with their vertebrae. Ten pairs of caudal ribs are observed in the holotype and at least eight in referred specimen. The anteriormost caudal ribs are slightly longer than the sacral ribs and have pointed distal ends. Notably, the caudal rib becomes a small round element from CdV6 (holotype) or 7 (WGSC V 1702).

Chevrons are developed in the caudal region. The first chevron appears at CdV5 in the holotype, while in the referred specimen the chevron could be recognized from CdV8 owing to its preservation. The bone is V-shaped, with an expanded proximal end. Chevron size decreases along the caudal vertebral column, disappearing in CdV28 in the holotype. The gastralia ossify in the

holotype but are mostly covered by dorsal ribs.

Appendicular skeleton. The pectoral girdle is partly exposed in both specimens (Fig. 3C–D). The clavicle is a wide L-shaped bone with a posterior process that contacts and overlaps the scapula, without the anterolateral prominence seen in *Diandongosaurus* or *Dianmeisaurus*[7,8]. The scapula contacts the clavicle with an anteromedial facet, and the coracoid posteriorly. It is relatively long and slender dorsally compared to the other elements of the pectoral girdle and to the robust scapula in *Dianmeisaurus gracilis*, *Diandongosaurus acutidentatus*, and *Hanosaurus hupehensis*[9-11]. There is a sign of slight ventral expansion of the scapula anteriorly, ventral to the clavicle overlap, but not as extensive as in *Wumengosaurus delicatomandibularis*, *Majiashanosaurus discocoracoidis*, and *Hongehesaurus longicaudalis*[12-14]. The scapula has a robust dorsal blade, extending posteriorly to the glenoid portion. Such a low shoulder could restrict the dorsal extension of the forelimb[1]. The coracoid is a large plate-like bone in this view, developing a rounded anteromedial margin, and forms the glenoid fossa with the scapula laterally. Though covered mostly in both holotype and referred specimen, a slightly waisted lateral borderline can be identified. The form of the coracoid is like that in *Majiashanosaurus discocoracoidis*[12], but lacks the stronger waist seen in Middle Triassic pachypleurosaurs like *Keichousaurus hui*, *Dianmeisaurus gracilis*, and *Diandongosaurus acutidentatus*[9,15,16]. It also lacks the small notching found in *Lariosaurus sanxianensis*[6]. Together, the scapula and the coracoid form the glenoid for articulation with the humerus at the dorsal blade laterally.

The humerus is curved, with a convex anterior margin and a concave posterior margin (Fig. 3G–H, K–L), a pattern characteristic of Sauropterygia[3]. Proximally, it expands slightly whereas the distal end expands further. A weakly developed deltopectoral crest exists on the anterior margin of the humerus. Both the ectepicondylar groove and the entepicondylar foramen are identifiable, near the proximal end, as in most pachypleurosaurids except *Diandongosaurus* and *Keichousaurus*[8]. The articular facet of the humerus with the pectoral girdle is smoothly curved. The radius is equal in length to the ulna, albeit slenderer, and together they overlap the posterior humerus (Fig. 3G–H, K–L). A more slenderly built radius than ulna is present in some pachypleurosaurs, like *Dianopachysaurus ding*[17], *Panzhousaurus rotundirostris*[18], and *Odoiporosaurus teruzzii*[2]. The ulna

has a more expanded proximal end compared to the distal end.

Six carpal ossifications are observed in both manus from the holotype (Fig. 3G–H), namely two large elements, the intermedium and the ulnare, and four small distal carpals, while the referred specimen only has the three carpal ossifications. The intermedium is oval-shaped, to some degree larger than the ulnare and situated between the distal ends of the radius and the ulna. The ulnare is a rounded element, located near the distal end of the ulna. Size increases from distal carpal 1 to 4 in the holotype, with distal carpal 4 intercalating between the intermedium and the ulnare. And distal carpal 1 in the right limb is nearer to the distal humerus, possibly due to taphonomic issues. Five metacarpals are all well preserved and exposed dorsally (Fig. 3G–H, K–L). Metacarpal 1 is flattened, and distinctly shorter and broader than the other metacarpals, like *Neusticosaurus* and many Alpine pachypleurosaurs[19], while metacarpal 3 is the longest (Table S1). Metacarpals 2–5 are rod-shaped with slightly expanded proximal and distal ends. The manual digits are deflected towards the ulnar side, and the digital interspace between digits 4 and 5 is the widest. Both forelimbs in the holotype have a phalangeal formula 2-3-4-5-3, with small triangle-shaped ungual phalanges. The extra phalangeal element in the referred specimen could belong to digit 5, of which the ungual phalanx is missing.

The ilium and the pubis are the only observable elements in the pelvic region (Fig. 3E–F). Viewed dorsally, the ilium develops a pointed posterior process, different to known pachypleurosaurid taxa, which mostly have poorly developed dorsal blades[9,17]. The ilium has a concave curve anterolaterally, forming the acetabular fossa. Most of the pubis is covered, with an exposed round lateral margin (Fig. 3E–F).

The femur is a long slender bone and is more weakly built than the humerus (Fig. 3I–J, M–N), like many other known eosauropterygians, for instance, *Keichousaurus hui*[4,20] and *Neusticosaurus pusillus*[19]. The bone has expanded proximal and distal ends, with the proximal end more expanded than the distal, and slightly curves posteriorly. Its distal end articulates with both the tibia and fibula laterally. The tibia is straight, nearly as robust as the femur, with a widened proximal end while its distal end barely expands (Fig. 3I–J, M–N). The tibia is overlapped by the fibula at their proximal ends: the latter is prominently more slender than the former though they are equal in length. The

fibula is curved, creating an interosseous space between the tibia and the fibula, and the proximal and distal ends of the fibula expand medially (Fig. 3I–J, M–N).

Six tarsal ossifications are preserved in both hindlimbs of the holotype: the astragalus, the calcaneum, and four distal tarsals, while the referred specimen only preserves three distal tarsals (Fig. 3I–J, M–N). The six tarsal ossifications in the holotype are a relatively high number among pachypleurosaurs (two in *Dianmeisaurus*, *Diandongosaurus*, *Wumengosaurus*[9,13,21]; three in *Hanosaurus*[10,22]). The astragalus and the calcaneum are rounded, flattened, and they articulate with the distal fibula, with the distal tarsal 4 positioned between them distally. The distal tarsals are tiny, rounded bones except distal tarsal 2, which is somewhat elongated, and the size increases from distal tarsal 1 to 4. As for the metacarpals, metatarsal 1 is the shortest and widest among the five metatarsals, and metatarsals 2 to 5 are rod-shaped with slightly expanded distal and proximal ends (Fig. 3 I-J, M-N). Length increases from metatarsal 1 to 4 and decreases in 5. The ungual phalanx of digit 4 on the left is missing when compared to the right pes; the phalangeal formula of the pes is 2-3-4-5-4, indicating no hyperphalangy in *C. xiangensis*. The metacarpals and metatarsals are short and rectangular, rather than slender hourglass-shaped like *Wumengosaurus* and *Panzhousaurus*[13,18]. Both the manual and pedal elements in *C. xiangensis* are flat and wide, without distinct expanded ends, showing a paddle-shaped overall outline.

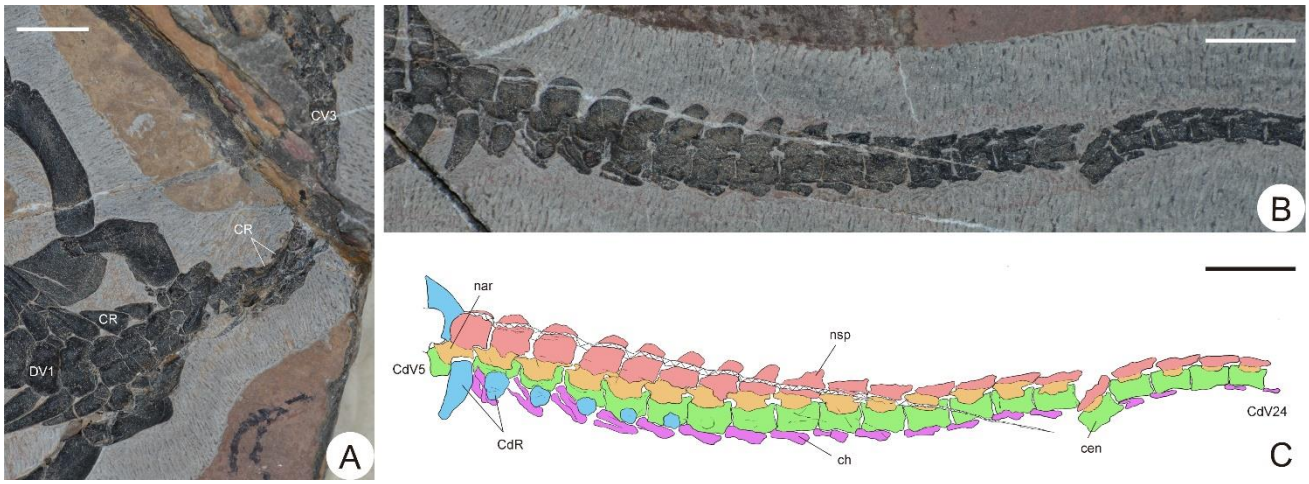

153

154 **Fig. S1. Selected postcranial parts of holotype**

155 **A.** Photograph of cervical vertebrae. **B.** Photograph of the caudal region, laterally exposed. **C.**

156 Interpretative drawing of the caudal region.. **Abbreviations: CdR**, caudal rib; **CdV**, caudal vertebra;

157 **cen**, centrum; **ch**, chevron; **CR**, cervical rib; **CV**, cervical vertebra;; **DV**, dorsal vertebra; **nar**, neural

158 arch; **nsp**, neural spine. Scale bar = 1 cm.

159

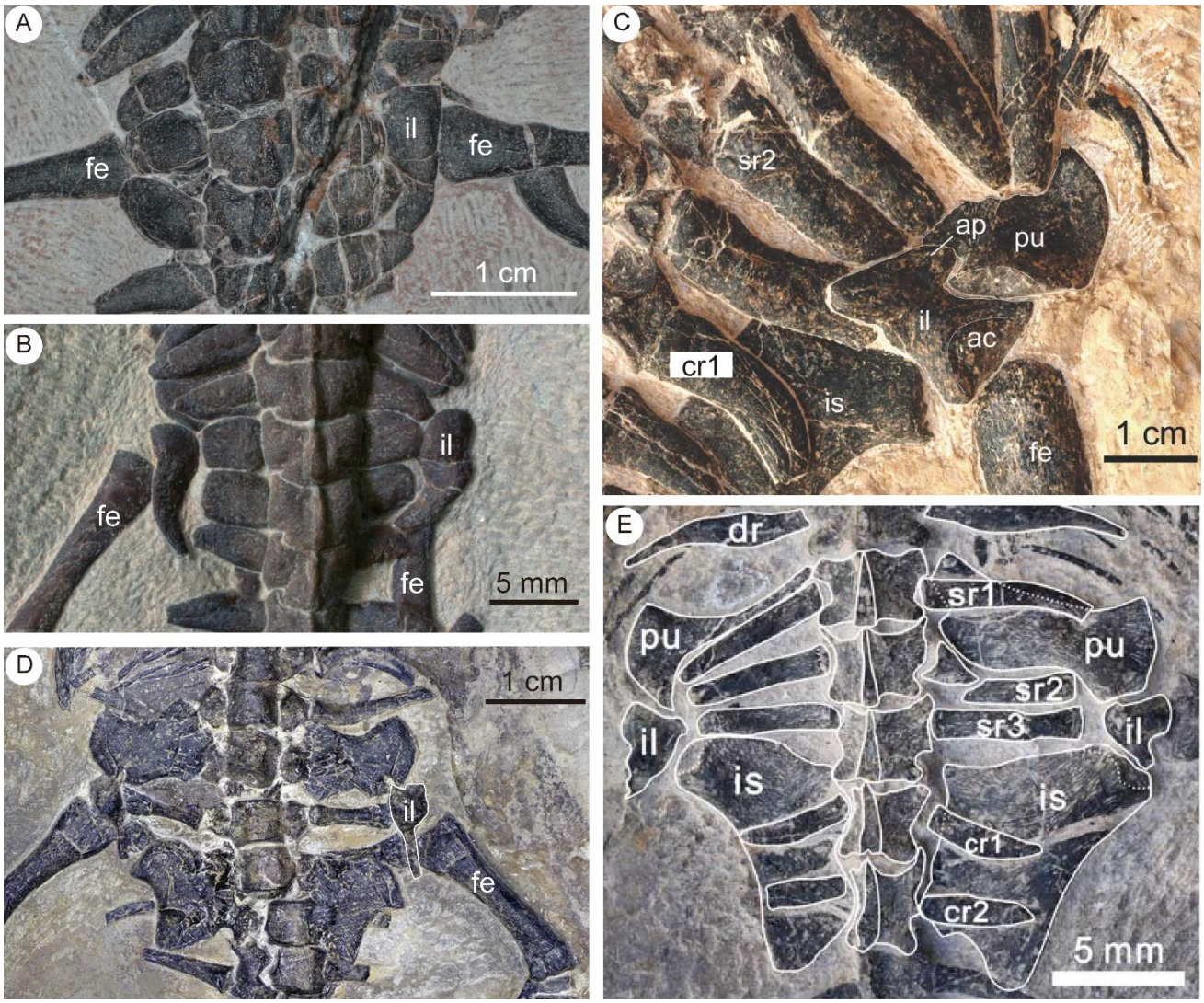

Fig. S2. Pelvic region of selected pachypleurosaurs

A. *Chusaurus xiangensis* (WGSC V 1901). **B.** *C. xiangensis* (WGSC V 1702). **C.** *Wumengosaurus delicatomandibularis*[12]. **D.** *Luopingosaurus imparilis*[49]. **E.** *Panzhousaurus rotundirostris*[18].

Abbreviations: ac, acetabulum; **ap**, anterior process of iliac blade; **cr**, caudal rib; **fe**, femur; **il**, ilium**; is,** ischium; **pu**, pubis; **sr,** sacral rib.

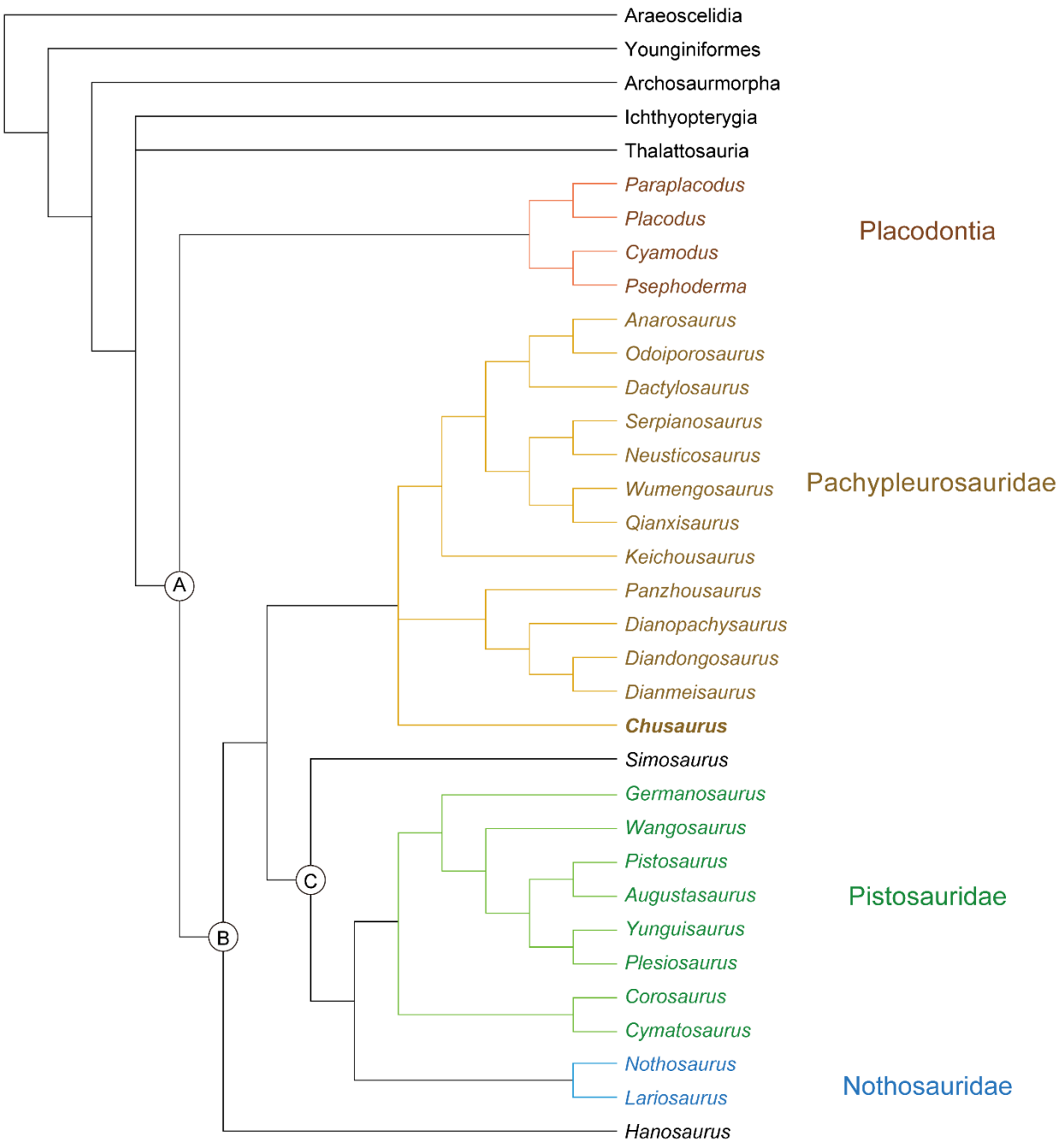

167

168 **Fig. S3. Strict consensus of four most parsimonious trees**

169 Bootstrap values ≥ 50% are labelled. Taxa are marked by different colours. **A.** Sauropterygia; **B.**

170 Eosauropterygia; **C.** Eusauropterygia.

171

172

173 **Table S1. Neck length/trunk length ratio of selected eosauropterygians**

Taxa	Species	Distribution	NL/TL	Reference
Basal eosauropterygian	*Hanosaurus hupehensis*	Early Triassic, South China	0.376	Wang et al.[9]
Pachypleurosauridae	*Chusaurus xiangensis*, gen. et sp. nov.	Early Triassic, South China	0.480	This study
Pachypleurosauridae	*Dwazisaurus brevis*	Middle Triassic, South China	0.930	Cheng et al.[23]
Pachypleurosauridae	*Diandongosaurus acutidentatus*	Middle Triassic, South China	0.765	Shang et al.[6]
Pachypleurosauridae	*Dianmeisaurus gracilis*	Middle Triassic, South China	0.693	Shang and Li[7]
Pachypleurosauridae	*Wumengosaurus delicatomandibularis*	Middle Triassic, South China	0.645	Jiang et al.[24]; Wu et al.[12]
Pachypleurosauridae	*Qianxisaurus chajiangensis*	Middle Triassic, South China	0.397	Cheng et al.[25]
Pachypleurosauridae	*Panzhousaurus rotundirostris*	Middle Triassic, South China	0.716	Jiang et al.[18]
Pachypleurosauridae	*Dianopachysaurus dingi*	Middle Triassic, South China	0.862	Liu et al.[17]
Pachypleurosauridae	*Keichousaurus hui*	Middle Triassic, South China	0.980	Lin and Rieppel[4]
Pachypleurosauridae	*Serpianosaurus mirigiolensis*	Middle Triassic, Monte San Giorgio	0.650	Rieppel and Greenwood[1]
Pachypleurosauridae	*Neusticosaurus pusillus*	Middle Triassic, Monte San Giorgio	0.730	Sander and Greenwood[19]
Pachypleurosauridae	*Dactylosaurus schroederi*	Middle Triassic, Poland	0.667	Sues and Carroll[26]

Nothosauridae	*Nothosaurus yangjuanensis*	Middle Triassic, South China	0.656	Yin et al.[27]
Nothosauridae	*Nothosaurus youngi*	Middle Triassic, South China	0.674	Ji et al.[28]
Nothosauridae	*Lariosaurus xingyiensis*	Middle Triassic, South China	0.600	Lin et al.[29]
Pistosauroidea	*Wangosaurus brevirostris*	Middle Triassic, South China	1.040	Ma et al.[30]
Pistosauroidea	*Yunguisaurus liae*	Middle Triassic, South China	0.960	Shang et al.[31]
Basal plesiosaurian	*Plesiosaurus dolichodeirus*	Early Jurassic, UK	1.885	Storrs[32]
Rhomaleosauridae	*Meyerasaurus victor*	Early Jurassic, Germany	0.848	Smith and Vincent[33]
Rhomaleosauridae	*Rhomaleosaurus thorntoni*	Early Jurassic, UK	0.949	Smith and Benson[34]
Pliosauridae	*Hauffiosaurus tomistomimus*	Early Jurassic, UK	1.640	Benson et al.[35]
Pliosauridae	*Hauffiosaurus zanoni*	Early Jurassic, Germany	1.060	Vincent[36]
Pliosauridae	*Luskhan itilensis*	Early Cretaceous, West Russia	0.483	Fischer et al.[37]
Pliosauridae	*Brachauchenius sp.*	Early Cretaceous, Colombia	0.427	Hampe[38]
Pliosauridae	*Sachicasaurus vitae*	Early Cretaceous, Colombia	0.312	Páramo-Fonseca et al.[39]
Pliosauridae	*Stenorhynchosaurus munozi*	Early Cretaceous, Colombia	0.513	Páramo-Fonseca et al.[40]

Elasmosauridae	*Jucha squalea*	Early Cretaceous, West Russia	1.981	Fischer et al.[41]
Elasmosauridae	*Albertonectes vanderveldei*	Late Cretaceous, Canada	3.618	Kubo et al.[42]
Polycotylidae	*Polycotylus latippinus*	Late Cretaceous, USA	0.797	O'Keefe and Chiappe[43]
Polycotylidae	*Mauriciosaurus fernandezi*	Late Cretaceous, Mexico	1.048	Frey et al.[44]
Cryptoclididae	*Tatenectes Laramiensis*	Late Jurassic, USA	0.672	O'Keefe et al.[45]
Cryptoclididae	PMO 224.248	Early Cretaceous, Norway	1.545	Roberts[46]
Cryptoclididae	*Kaiwhekea katiki*	Late Cretaceous, New Zealand	1.906	Cruickshank and Fordyce[47]
Pistosauroidea	*Corosaurus alcovensis*	Early Triassic, USA	0.500	Gutarra et al.[48]

174

175

176 **Table S2. Selected measurements (in mm) of WGSC V 1901 and WGSC V 1702**

	WGSC V 1901	WGSC V 1702
Skull and mandible		
Skull length	29.40	-
Length from tip of snout to anterior margin of orbit	11.58	-
Length from posterior margin of orbit to end of skull table	10.47	-
Maximum length of orbit	7.35	-
Width of orbit	4.75	-
Postcranial skeleton		
Length of cervical vertebral column	53.10	~35.20
Trunk length	103.70	~77.20
Length of preserved caudal vertebral column	166.20	58.12
Total length of scapular	13.27	-
Length of left humerus	23.08	15.60
Proximal width of left humerus	5.71	6.19
Distal width of left humerus	6.42	4.69
Minimal width of left humerus	4.95	4.44
Length of left ulna	12.91	7.23
Minimum width of left ulna	2.32	1.50
Length of left radius	12.47	7.56
Minimum width of left radius	2.05	1.55
Length of left metacarpal 1	2.94	1.84
Length of left metacarpal 2	4.09	3.00
Length of left metacarpal 3	5.12	3.07
Length of left metacarpal 4	4.12	2.88
Length of left metacarpal 5	3.25	2.16
Maximum width of left intermedium	3.23	1.44
Maximum width of left ulnare	1.68	0.91

Total length of ilium	11.22	7.56
Length of left femur	17.52	13.80
Proximal width of left femur	4.85	-
Distal length of left femur	3.40	2.35
Minimal width of left femur	2.57	1.80
Length of left tibia	12.33	8.40
Length of left fibular	12.70	8.61
Length of left metatarsal 1	3.60	2.40
Length of left metatarsal 2	5.95	4.08
Length of left metatarsal 3	6.60	4.68
Length of left metatarsal 4	6.70	4.69
Length of left metatarsal 5	5.45	3.96
Maximum width of left calcaneum	3.73	2.04
Maximum width of left astragalus	4.12	2.23

177 **(**Measurements of WGSC V 1702 are converted from the photos**)**

178

179 **Table S3. Neck length/trunk length ratio for R**

Species	Group	NeckL/TrunkL	FAD	LAD
Hanosaurus	Eosauropterygian	0.375	251.2	247.2
Dawazisaurus_brevis	Pachypleurosaur	0.93	247.2	242
Diandongosaurus	Pachypleurosaur	0.765	247.2	242
Dianmeisaurus	Pachypleurosaur	0.693	247.2	242
Wumengosaurus	Pachypleurosaur	0.645	247.2	242
Qianxisaurus	Pachypleurosaur	0.397	242	237
Serpianosaurus	Pachypleurosaur	0.65	242	242
Neusticosaurus	Pachypleurosaur	0.73	242	237
Dactylosaurus	Pachypleurosaur	0.667	247.2	242
Panzhousaurus	Pachypleurosaur	0.716	247.2	242
Dianopachysaurus	Pachypleurosaur	0.862	247.2	242
Keichousaurus	Pachypleurosaur	0.98	242	237
Chusaurus	Pachypleurosaur	0.48	248.5	247.2
Nothosaurus	Nothosaur	0.656	247.2	242
Nothosaurus_youngi	Nothosaur	0.674	242	237
Lariosaurus	Nothosaur	0.6	242	237
Wangosaurus	Pistosaur	1.04	242	237
Yunguisaurus	Pistosaur	0.96	242	237
Corosaurus	Pistosaur	0.5	251.2	247.2
Plesiosaurus	Plesiosaur	1.885	195.31	190.82

180

181

Dataset S1. Matrix for phylogenetic analysis

Araeoscelidia

00110000000000000000110101100000000020(01)000000?000000000000000010000000020(01)0
00000000000?00000000000?0000100-0100000001000000?10100000000000100000110000

Younginiformes

00110000000000000000110100100000000020100000 1?0000000000000000?1000000001-
000000000000000000000000000?1000(01)00-
0100000(02)01(01)0000?00100010100001000011000 0

Archosaurmorph

01110000000100000000 1(01)000110000(01)01(23)0202100(01)11?001(01)000000000001(01)0000
00001-0(03)(02)0000(01)000000?0100000000001000 0?0-
0100000011(12)0000?00100(01)101001010001110001

Ichthyopterygia

000120?1?2010?0??0(01)0?0(012)(01)???000(01)0(13)?(03)0(02)02100011?00001(012)?00(01)002
?00001?0(01)0001?030100000?????0???0???????0110?(01)0??2(01)??11131?(12)???00?4???(01)01
2?0?????????????

Thalattosaur

001(01)20?(01)?(12)00(01)?1??000?(01)?1???000000?(03)02021001?1?00111??00100(02)0??11000
00(01)01?03000?000???00????0??00?000100?100?00??111?(01)?2???00?0?000111(01)11?????????
???

Paraplacodus

00001?0002??01?00?1?1??10000001??0?000210?110?0???0???1?01????11210111 01?01000?010?1
1100?0100?0001111000??1?1010010?11200011031012101 11?000??0??0?00

Placodus

01021100011101 00100001(01)11010000010(02)0000000100111100110001110110001020011101?0200
00010001100101000000101100011 0?00101100102000030100021012010010011100 02

Cyamodus

1202110001(01)0000000001(01)0101000102003000010000100011100011011 0?10200110 01?0200

0001000?1000000??00010(01)?000?20?0??0110??1200002?100120??????000001?0?01

Psephoderma

12022100020011101000100101000102103000010100100011100?100111??????011201?0200??0?0?

??10?00000?000101??????0?10100102120000??100121011110000001?0?01

Anarosaurus

000100000000000000000101000000100000202000??11002011 0?000021?00001100011?030001100

010001000000(01)100001110(01)21(01)2000011101100002021111112001010010100?00

Dactylosaurus

001100000000000100000101000000100000202000??11002011 0?000021?10001000001?030101100

00000100000?0100000110??1020?00100011000021310 01?1?101010000100000

Serpianosaurus

001100000000000000000101000000100000202000011100200 10?000021?10001000001?03010110

0010001000(01)00(01)100000100021020(01)0(01)11101(12)000030311111122110100001000 00

Neusticosaurus

00(01)10000000000(01)00000(01)10100100010000020200001110020010?000021?(01)0001000001

?03010110001000100010 0(01)110000100(01)21020(01)0(01)1(01)(01)0110000(23)(0 1)310(0

1)1112(1 2)11010000100000

Odoiporosaurus

001100000000001000000111000000100000202000011?00?01???????????00??100000?0?010110??

?000100000???0000??10????2010011101100 00??3102111 2????100?0??0?00

Hanosaurus

000200??0?010?00000101 0?000000?20?30202000????00?1110?????????????00??01?0?010???????

????0?1????00101?00????20??????????????????00101011 11??00?0??????

Wumengosaurus

000120000100000000000101000001100000202100011?00?00????0?021??00?0100001?030101 0?0

010001001100010000001002 1120(01)011100110000210001211 211?100?0100002

Panzhousaurus

0012000000000000000000010000000(12)00302020001l1?0020010???002???00?1000001?030??11

000000010001(01)0010000???0??10201011110220000031111112(01)111000?0100100

Dianopachysaurus

0012000000000000000101110000001200302020?0?0?00?01?0?????????00?100000000?????1?0?

0?00?00010001?0000?10??10?000?11102200201121?1?1122111100?01?0?01

Diandongosaurus

0001000000000000001101110000001110302020000111012101O?000020?0100100011O003?0010?0

0000010000?00100001112021120001113111000030?1111112201l?1000100100

Dianmeisaurus

0002000002000000000001110000001(12)0030202000??1?0021010?000020?0100100001O003?001

0?000?00?000100110000111202112000111102200002021111112201110000100101

Keichousaurus

0012000000001010000101010000000200302021000l0?0020010?000021?01001000(01)00?030101

1000000010001000100000110121020l0(01)(01)11121102000310011121010100011010O1

Qianxisaurus

0011100001000000000001010000001000302021000l1?0020010?????????00?0100001?0?010?1?00

1000?000010110000l?10??112000?11301100003031?11112211010O?0100?00

Chusaurus

(01)0??010??????????????????????0??????????????????????0???00?01?????0??00001?03?0?11?0?0?

00??0111001???00?10??00201011110120000(02)001???01?(01)010????01?10?1

Simosaurus

000200010000001(01)00011(01)(01)11000000210101020000100012110020000201101001001O1?2

3100100002000110000?000010011111102010011111200102021101112111010000??1002

Germanosaurus

0002111101000011100011111000l?01111110200???0?00?1??????0?????????000111?13???????????

??110????????

Nothosaurus

000(12)1101(01)(01)000(01)(01)(01)10011(01)0(12)(01)(01)(01)(01)(01)002(12)(01)(12)1102000(0
1)100012(01)(01)1120100(02)0110120000110(012)231101(01)000(01)00010(01)00(01)1(012)1001
0(01)(01)10(01)(12)1020(01)(01)(01)01(01)012(01)(01)(01)0(12)0211(01)110(12)1110(01)(01)0111
01(1)01

Lariosaurus

00021(01)(01)11(02)(01)00(01)(01)(01)10(01)11(01)0(12)0(01)0(01)(01)002(012)(01)(012)1(01)02
00011000120(01)1120100(02)0?(01)0120000(01)10(01)(12)31101100010001000100(01)(01)01(12)100(0
1)0(01)(01)(01)0(02)21(01)(02)0(01)(01)(01)11(01)(01)(01)(012)1(01)(01)00031(01)(01)11(01)(12)
(012)(01)10(01)(01)(01)(01)1101100

Corosaurus

00001001?2000000000011110000??000100000200l??0101111O????0000??1110000110013000100000
1001110101100001010101111200011100120000201001110110101?0?1100?01

Cymatosaurus

000211?10200101100(01)011(12)10000000(12)(23)000002001l?0?0(01)11100?0100001l??200001
10013100l?0???0011?0?00????000???????????000000?????0????011101????1001?100?01

Wangosaurus

000210010200001000011001000001023110122000110?0111010?????????11?000?11002???01001?
?0001?0000110??????10???0?000?11001?0???00?????1??0110110?01001??

Pistosaurus

000021010l?02?1?0100112100000102312012201?1?0112111???0?0000??????00011001310110001
?0011?1000????0?0??????113?00?1131220001??0????11??????1???100?1(12)

Augustasaurus

000021010l??2?1?0100112100000100312012201?1?00121112010110000??0120000010023101?001
111011?000????00?00?00??103??02113102000120???????????0?010100???

Yunguisaurus

000011010200101000011011000O??00312012201?1?0112110?020?10200011200001100l3(01)1010
02?110?1?1100?0000000??0??113110111311210010040021012000011000100111

Plesiosaurus

292 0000000102??2?1?000010010100010031201220101?011211020101102O??0100000010013(01)010

293 00201101101000?1010?00100??1031101103112102000400210120000?1010010111

294

References

1. Rieppel, O. (1989). A new pachypleurosaur (Reptilia: Sauropterygia) from the Middle Triassic of Monte San Giorgio, Switzerland. Philos. Trans. R. Soc. Lond. Ser. B-Biol. 323, 1-73.
2. Renesto, S., Binelli, G., and Hagdorn, H. (2014). A new pachypleurosaur from the Middle Triassic Besano Formation of Northern Italy. N. Jb. Geol. Paläontol. Mh. 271, 151-168.
3. Rieppel, O. (2000). Sauropterygia I: Placodontia, Pachypleurosauria, Nothosauroidea, Pistosauroidea. Encycl. Paleoherpetol. 12, 1-134.
4. Lin, K., and Rieppel, O. (1998). Functional morphology and phylogeny of *Keichousaurus hui* (Sauropterygia, Reptilia). Fieldiana: Geol. new Ser. 39, 1-35.
5. Klein, N. (2012). Postcranial morphology and growth of the pachypleurosaur *Anarosaurus heterodontus* (Sauropterygia) from the Lower Muschelkalk of Winterswijk, The Netherlands. Paläont. Z. 86, 389-408.
6. Li, Q., and Liu, J. (2020). An Early Triassic sauropterygian and associated fauna from South China provide insights into Triassic ecosystem health. Commun. Biol. 3, 63. 10.1038/s42003-020-0778-7.
7. Shang, Q., Wu, X., and Li, C. (2011). A new eosauropterygian from Middle Triassic of eastern Yunnan Province, southwestern China. Vet. PalAsiat. 49, 155-171.
8. Shang, Q., and Li, C. (2015). A new small-sized eosauropterygian (Diapsida: Sauropterygia) from the Middle Triassic of Luoping, Yunnan, southwestern China. Vet. PalAsiat. 53, 265-280.
9. Shang, Q., Li, C., and Wu, X. (2017). New information on *Dianmeisaurus gracilis* Shang & Li, 2015. Vet. PalAsiat. 55, 145-161.
10. Wang, W., Shang, Q., Cheng, L., Wu, X.-C., Li, C. (2022). Ancestral body plan and adaptive radiation of sauropterygian marine reptiles. iScience, 25, 105635. 10.1016/j.isci.2022.105635.
11. Sato, T., Cheng, Y.-N., Wu, X.-C., and Shan, H.-Y. (2014). *Diandongosaurus acutidentatus* Shang, Wu & Li, 2011 (Diapsida: Sauropterygia) and the relationships of Chinese eosauropterygians. Geol. Mag. 151, 121-133.

12. Jiang, D.-Y., Motani, R., Tintori, A., Rieppel, O., Chen, G.-B., Huang, J.-D., Zhang, R., Sun, Z.-Y., and Ji, C. (2014). The Early Triassic eosauropterygian *Majiashanosaurus discocoracoidis*, gen. et sp. nov. (Reptilia, Sauropterygia), from Chaohu, Anhui Province, People's Republic of China. J. Vert. Paleontol. 34, 1044-1052.
13. Wu, X.-C., Cheng, Y.-N., Li, C., Zhao, L.-J., and Sato, T. (2011). New information on *Wumengosaurus delicatomandibularis* Jiang et al., 2008 (Diapsida: Sauropterygia), with a revision of the osteology and phylogeny of the taxon. J. Vert. Paleontol. 31, 70-83.
14. Xu, G.-H., Ren, Y., Zhao, L.-J., Liao, J.-L., and Feng, D.-H. (2022). A long-tailed marine reptile from China provides new insights into the Middle Triassic pachypleurosaur radiation. Sci. Rep. 12, 7396. 10.1038/s41598-022-11309-2.
15. Xue, Y.-F., Jiang, D.-Y., Motani, R., Rieppel, O., and Sun, Y.-L. (2013). New information on sexual dimorphism and allometric growth in *Keichousaurus hui*, a pachypleurosaur from the Middle Triassic of Guizhou, South China. Acta Palaeontol. Pol. 60, 681-687.
16. Liu, X.-Q., Lin, W.-B., Rieppel, O., Sun, Z.-Y., Li, Z.-G., Hao, L., and Jiang, D.-Y. (2015). A new specimen of *Diandongosaurus acutidentatus* (Sauropterygia) from the Middle Triassic of Yunnan, China. Vet. PalAsiat. 53, 1-5.
17. Liu, J., Rieppel, O., Jiang, D.-Y., Aitchison, J.C., Motani, R., Zhang, Q.-Y., Zhou, C.-Y., and Sun, Y.-Y. (2011). A new pachypleurosaur (Reptilia: Sauropterygia) from the lower Middle Triassic of southwestern China and the phylogenetic relationships of Chinese pachypleurosaurs. J. Vert. Paleontol. 31, 292-302.
18. Jiang, D.-Y., Lin, W.-B., Rieppel, O., Motani, R., and Sun, Z.-Y. (2019). A new Anisian (Middle Triassic) eosauropterygian (Reptilia, Sauropterygia) from Panzhou, Guizhou Province, China. J. Vert. Paleontol. 38, 1044-1052.
19. Sander, P.M. (1989). The pachypleurosaurids (Reptilia: Nothosauria) from the Middle Triassic of Monte San Giorgio (Switzerland) with the description of a new species. Philos. Trans. R. Soc. Lond. Ser. B-Biol. 325, 561-666.
20. Young, C.-c. (1958). On the new Pachypleurosauroidea from Keichow,South-West China. Vet. PalAsiat. 2, 69–81.

21. Liu, Q.-L., Yang, T.-L., Cheng, L., Benton, M.J., Moon, B.C., Yan, C.-B., An, Z.-H., and Tian, L. (2021). An injured pachypleurosaur (Diapsida: Sauropterygia) from the Middle Triassic Luoping Biota indicating predation pressure in the Mesozoic. Sci. Rep. 11, 21818.
22. Rieppel, O. (1998). The systematic status of *Hanosaurus hupehensis* (Reptilia, Sauropterygia) from the Triassic of China. J. Vert. Paleontol. 18, 545-557.
23. Cheng, Y.-N., Wu, X., Sato, T., and Shan, H.-Y. (2016). *Dawazisaurus brevis*, a new eosauropterygian From the Middle Triassic of Yunnan, China. Acta Geol. Sin. 90, 401-424.
24. Jiang, D.-Y., Rieppel, O., Motani, R., Hao, W.-C., Sun, Y.-L., Schmitz, L., and Sun, Z.-Y. (2008). A new Middle Triassic eosauropterygian (Reptilia, Sauropterygia) from southwestern China. J. Vert. Paleontol. 28, 1055-1062.
25. Cheng, Y.-N., Wu, X.-C., Sato, T., and Shan, H.-Y. (2012). A new eosauropterygian (Diapsida, Sauropterygia) from the Triassic of China. J. Vert. Paleontol. 32, 1335-1349.
26. Sues, H.-D., and Carroll, R.L. (1985). The pachypleurosaurid *Dactylosaurus schroederi* (Diapsida: Sauropterygia). Can. J. Earth Sci. 22, 1602-1608.
27. Yin, C., Hao, W., Sun, Z., Sun, Y., and Jiang, D. (2014). New material of *Nothosaurus yangjuanensis* from the Middle Anisian (Middle Triassic) of Guizhou Province, Southwestern China. Acta Sci. Nat. Univ. Pekin. 50, 467-475.
28. Ji, C., Jiang, D.-Y., Rieppel, O., Motani, R., Tintori, A., and Sun, Z.-Y. (2014). A new specimen of *Nothosaurus youngi* from the Middle Triassic of Guizhou, China. J. Vert. Paleontol. 34, 465-470.
29. Lin, W.-B., Jiang, D.-Y., Rieppel, O., Motani, R., Ji, C., Tintori, A., Sun, Z.-Y., and Zhou, M. (2017). A new specimen of *Lariosaurus xingyiensis* (Reptilia, Sauropterygia) from the Ladinian (Middle Triassic) Zhuganpo Member, Falang Formation, Guizhou, China. J. Vert. Paleontol. 37, e1278703. 10.1080/02724634.2021.1901730.
30. Ma, L.-T., Jiang, D.-Y., Rieppel, O., Motani, R., and Tintori, A. (2015). A new pistosauroid (Reptilia, Sauropterygia) from the late Ladinian Xingyi marine reptile level, southwestern China. J. Vert. Paleontol. 35, e881832. 10.1080/02724634.2014.881832.
31. Shang, Q.H., Sato, T., Li, C., and Wu, X.C. (2017). New osteological information from a

'juvenile' specimen of *Yunguisaurus* (Sauropterygia; Pistosauroidea). Palaeoworld 26, 500-509.

32. Storrs, G.W. (1997). Chapter 6 - Morphological and taxonomic clarification of the genus *Plesiosaurus*. In Ancient Marine Reptiles, J.M. Callaway and E.L. Nicholls, eds. (San Diego: Academic Press), pp. 145-190.

33. Smith, A.S., and Vincent, P. (2010). A new genus of pliosaur (Reptilia: Sauropterygia) from the Lower Jurassic of Holzmaden, Germany. Paleontology 53, 1049-1063.

34. Smith, A.S., and Benson, R.B.J. (2014). Osteology of *Rhomaleosaurus thorntoni* (Sauropterygia: Rhomaleosauridae) from the Lower Jurassic (Toarcian) of Northamptonshire, England. Monogr. Palaeontogr. Soc. 168, 1-40.

35. Benson, R.B.J., Bates, K.T., Johnson, M.R., and Withers, P.J. (2011). Cranial anatomy of *Thalassiodracon hawkinsii* (Reptilia, Plesiosauria) from the Early Jurassic of Somerset, United Kingdom. J. Vert. Paleontol. 31, 562-574.

36. Vincent, P. (2011). A re-examination of *Hauffiosaurus zanoni*, a pliosauroid from the Toarcian (Early Jurassic) of Germany. J. Vert. Paleontol. 31, 340-351.

37. Fischer, V., Benson, R.B.J., Zverkov, N.G., Soul, L.C., Arkhangelsky, M.S., Lambert, O., Stenshin, I.M., Uspensky, G.N., and Druckenmiller, P.S. (2017). Plasticity and convergence in the evolution of short-necked plesiosaurs. Curr. Biol. 27, 1667-1676, e1663.

38. Hampe, O. (2005). Considerations on a *Brachauchenius* skeleton (Pliosauroidea) from the lower Paja Formation (late Barremian) of Villa de Leyva area (Colombia). Mitt. Mus. Nat. Berl. – Geowiss. Reihe 8, 37-51.

39. Paramo-Fonseca, M.E., Benavides-Cabra, C.D., and Gutierrez, I.E. (2018). A new large pliosaurid from the Barremian (Lower Cretaceous) of Sachica, Boyaca, Colombia. Earth Sci. Res. J. 22, 223-238.

40. Páramo-Fonseca, M.E., Gómez-Pérez, M., Noé, L.F., and Etayo-Serna, F. (2016). *Stenorhynchosaurus munozi*, gen. et sp. nov. a new pliosaurid from the Upper Barremian (Lower Cretaceous) of Villa de Leiva, Colombia, South America. Rev. Acad. Colomb. Cienc. Exactas, Fis. Nat. 40, 84-103.

41. Fischer, V., Zverkov, N.G., Arkhangelsky, M.S., Stenshin, I.M., Blagovetshensky, I.V., and Uspensky, G.N. (2020). A new elasmosaurid plesiosaurian from the Early Cretaceous of Russia marks an early attempt at neck elongation. Zool. J. Lin. Soc. 192, 1167-1194.
42. Kubo, T., Mitchell, M.T., and Henderson, D.M. (2012). *Albertonectes vanderveldei*, a new elasmosaur (Reptilia, Sauropterygia) from the Upper Cretaceous of Alberta. J. Vert. Paleontol. 32, 557-572.
43. O'Keefe, F.R., and Chiappe, L.M. (2011). Viviparity and K-selected life history in a Mesozoic marine plesiosaur (Reptilia, Sauropterygia). Science 333, 870-873.
44. Frey, E., Mulder, E.W.A., Stinnesbeck, W., Rivera-Sylva, H.E., Padilla-Gutierrez, J.M., and Gonzalez-Gonzalez, A.H. (2017). A new polycotylid plesiosaur with extensive soft tissue preservation from the early Late Cretaceous of northeast Mexico. Bol. Soc. Geol. Mex. 69, 87-134.
45. O'Keefe, F.R., Street, H.P., Wilhelm, B.C., Richards, C.D., and Zhu, H. (2011). A new skeleton of the cryptoclidid plesiosaur *Tatenectes laramiensis* reveals a novel body shape among plesiosaurs. J. Vert. Paleontol. 31, 330-339.
46. Roberts, A.J. (2017). Taxonomy, palaeobiogeography and relationships of cryptoclidid plesiosaurs from the Slottsmøya Member, Agardhfjellet Formation, central Spitsbergen. In the Department of Ocean and Earth Science, Volume Doctor of Philosophy. (the University of Southampton), p. 442.
47. Cruickshank, A.R.I., and Fordyce, R.E. (2002). A new marine reptile (Sauropterygia) from New Zealand: further evidence for a Late Cretaceous austral radiation of cryptoclidid plesiosaurs. Paleontology 45, 557-575.
48. Gutarra, S., Stubbs, T.L., Moon, B.C., Palmer, C., and Benton, M.J. (2022). Large size in aquatic tetrapods compensates for high drag caused by extreme body proportions. Commun. Biol. 5, 380. 10.1038/s42003-022-03322-y.
49. Xu, G.-H., Shang, Q.-H., Wang, W., Ren, Y., Lei, H., Liao, J.L., Zhao, L.J. , Li, C. (2023). A new long-snouted marine reptile from the Middle Triassic of China illuminates pachypleurosauroid evolution. Sci. Rep. 13, 16. 10.1038/s41598-022-24930-y
